# A comparison of two headless compression screws for operative treatment of scaphoid fractures

**DOI:** 10.1186/1749-799X-6-27

**Published:** 2011-06-07

**Authors:** Ruby Grewal, Joseph Assini, David Sauder, Louis Ferreira, Jim Johnson, Kenneth Faber

**Affiliations:** 1 Hand and Upper Limb Centre, St. Joseph's Health Care, 268 Grosvenor St., London, ON, N6A 4L6, Canada

## Abstract

**Purpose:**

The purpose of this study was to compare the interfragmentary compression force across a simulated scaphoid fracture by two commonly used compression screw systems; the Acutrak 2 Standard and the 3.0 mm Synthes headless compression screw.

**Methods:**

Sixteen (8 pairs; 6 female, 2 male) cadaver scaphoids were randomly assigned to receive either the Acutrak 2 or Synthes screw with the contralateral scaphoid designated to receive the opposite. Guide wires were inserted under fluoroscopic control. Following transverse osteotomy, the distal and proximal fragments were placed on either side of a custom load cell, to measure interfragmentary compression. Screws were placed under fluoroscopic control using the manufacturer's recommended surgical technique. Compressive forces were measured during screw insertion. Recording continued for an additional 60s in order to measure any loss of compression after installation was complete. The peak and final interfragmentary compression were recorded and paired t-tests performed.

**Results:**

The mean peak compression generated by the Acutrak 2 Standard was greater than that produced by the Synthes compression screw (103.9 ± 33.2 N vs. 88.7 ± 38.6 N respectively, p = 0.13). The mean final interfragmentary compression generated by the Acutrak 2 screw (68.6 ± 36.4 N) was significantly greater (p = 0.04) than the Synthes screw (37.2 ± 26.8 N). Specimens typically reached a steady state of compression by 120-150s after final tightening.

**Conclusion:**

Peak interfragmentary compression observed during screw installation was similar for both screw systems. However, the mean interfragmentary compression generated by the Acutrak 2 Standard was significantly greater. Our study demonstrates that the Synthes headless compression screw experienced a greater loss of interfragmentary compressive force from the time of installation to the final steady state compression level. The higher post-installation compression of the Acutrak 2 Standard may be attributable to the greater number of threads throughout the entire length of the screw. The clinical significance of these results, are, at this point uncertain. We do demonstrate that a fully threaded design offers a more reliable compression that may translate to more predictable bony union.

## Introduction

The scaphoid is commonly injured, and is one of the most frequently fractured bones of the wrist [1]. Treatment options include cast immobilization, closed reduction and percutaneous pinning or open reduction internal fixation [1]. In recent years, compression screws have been increasingly used for treatment of this injury. Interfragmentary compression and stable fixation is important to fracture union [1], and an advantage of internal fixation. Although the optimum force required to produce osseous union in vivo remains unknown, it is believed that greater interfragmentary compression promotes more predictable healing [2,3].

Surgical fixation of the scaphoid is the accepted standard of care for the treatment of nonunions, delayed unions and displaced fractures [1,4-6]. Recently surgical fixation has been advocated as a viable treatment option for acute undisplaced scaphoid fractures [7-12] particularly when an accelerated return to function is desired [11,12]. Evaluations of the Acutrak screw report that patients with undisplaced scaphoid fractures treated with percutaneous fixation have a faster return to work and sports [13] and require less time for bony union when compared to cast immobilization [14]. These findings are relevant for younger active patients who sustain the majority of scaphoid fractures [1]. Studies comparing the Herbert screw to cast immobilization did not identify any long term radiographic or clinical benefits to surgical fixation versus casting [14] and longer term follow up did not demonstrate significant benefits with surgical treatment [15]. Given the controversy that exists around operative fixation of the acute minimally displaced scaphoid fracture, surgeons must look closely at patient factors prior to recommending surgery or selecting a screw system.

A variety of internal fixation systems are commercially available and have been studied for the treatment of scaphoid fractures. In an *in-vitro *study, the Acutrak Standard screw (Acumed^®^, Hillsbro, OR, USA) provided more compression than the Bold screw (Wright Medical Technology, Memphis TN) and Acutrak Mini screw (Acumed^®^, Hillsbro, OR, USA) [16]. As well, the Synthes 3.0 mm headless screw (Synthes Inc^®^, Westchester, PA, USA) provided reliable compression in a cadaveric model [17]. The purpose of this study was to compare the magnitude of compression between the 3.0 mm Synthes headless compression screw and the Acutrak 2 Standard screw (Acumed^®^, Hillsbro, OR, USA). We hypothesized that the Acutrak 2 Standard screw would provide more reliable compression when compared to the Synthes headless compression screw in a cadaveric model.

## Methods

Eight paired (6 female, 2 male) fresh frozen cadaveric scaphoids with a mean age of 75 (range 47-87) years were tested. Each scaphoid was carefully harvested, stripped of soft tissue, examined with fluoroscopy to ensure the absence of abnormalities and frozen at -20°C. Eight scaphoids were randomly assigned to receive the Synthes 3.0 mm headless compression screw, while the 8 remaining contralateral scaphoids received the Acutrak 2 screw.

**Figure 1:**
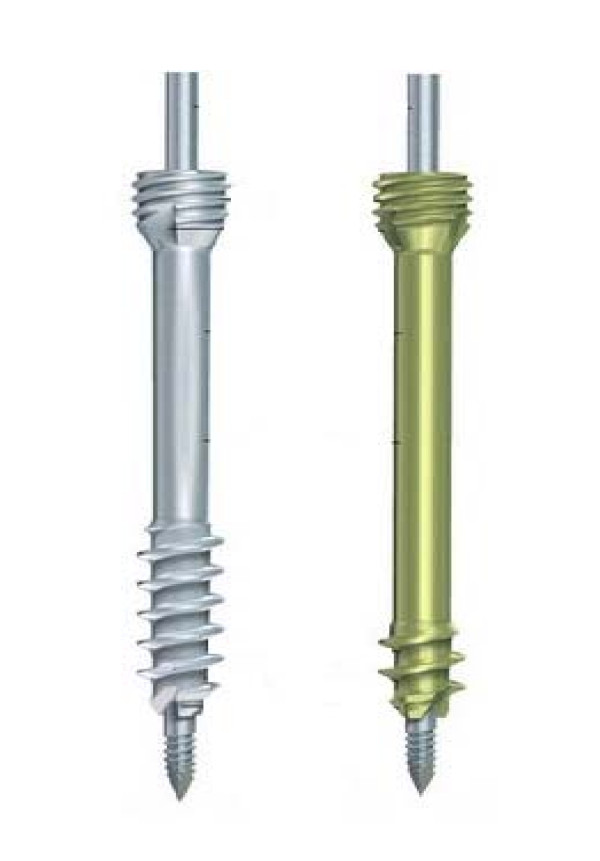
**The Synthes 3.0 mm headless compression screw consists of a threadless central shaft with threads of differential pitch at either end promoting fracture compression**. Screws are available in long and short threaded designs, as shown on the left and right respectively.

**Figure 2:**
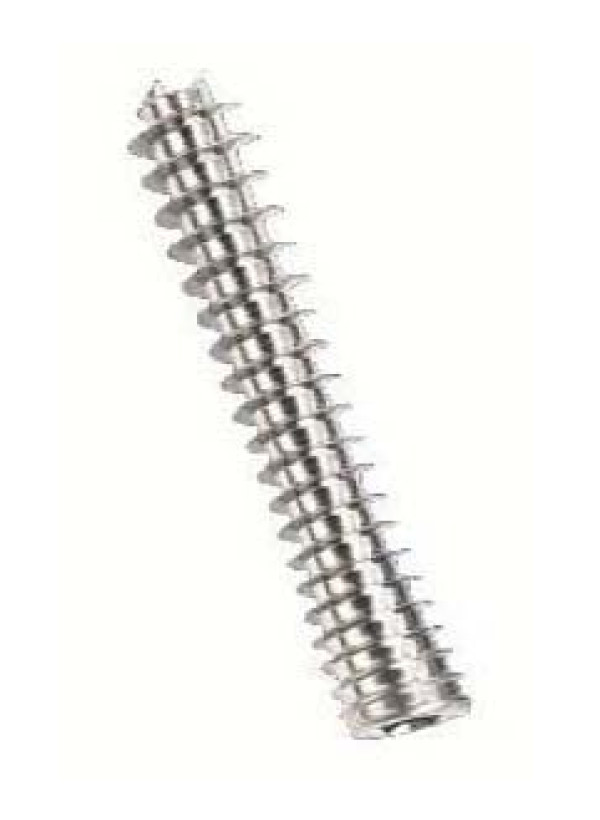
**The Acutrak 2 screw has a head diameter of 4.1 mm and a tip diameter of 4.0 mm**. The variable thread pitch causes the screw to advance through the two bone fragments at different rates, causing gradual compression.

The Synthes screw is composed of titanium and is a headless design. It consists of a threadless central shaft with threads of differential pitch at either end promoting fracture compression. The proximal threads are dual to increase bone purchase and the screw is available in long (40% of screw length threaded) and short (20% of screw length threaded) configurations. Available sizes range from 10-40 mm for long screws and 16-40 mm for short screws (Figure 1). All screws tested in our study were long threaded screws. The Acutrak 2 Standard headless compression screw has an hourglass shape and is composed of titanium alloy. Head diameter is 2.8 mm while the tail diameter is 4.7 mm. The variable thread pitch design, which is wider at the distal end, causes the screw to engage the two bone fragments at different rates causing gradual compression of the fracture as the screw is advanced. Screws are available in sizes from 16 mm to 30 mm in 2 mm increments (Figure 2). The 3.0 mm Synthes compression screw has been shown to provide reliable interfragmentary compression [17,18] while the Acutrak 2 is a relatively new addition to the market.

**Figure 3:**
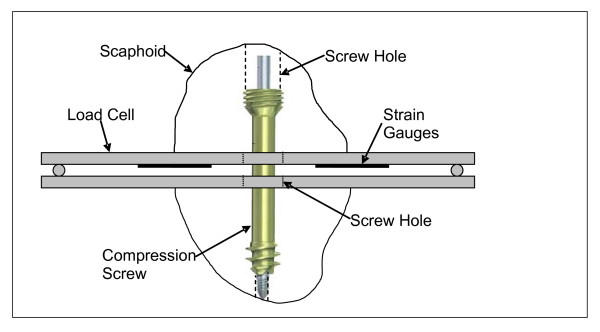
**Load Cell used to measure interfragmentary compression**. The strain gauge based load cell consisted of two parallel beams interposed in the fracture site. A central hole in the load cell accommodated the compression screw. Shown with a Synthes screw.

Interfragmentary compression was measured using a custom load cell that was interfaced with a data recording computer. This methodology and instrumentation has been previously described [17]. The load cell consisted of two parallel beams interposed in the fracture site and had an overall thickness of 5 mm (Figure 3). A central hole in the load cell accommodated the compression screw. One of the beams was instrumented with strain gauges (EA-06-062AQ-350, Micromeasurements, Measurement Group Inc., Raleigh, NC) in a two full-bridge configuration with one full-bridge on either side of the central hole. The output of the independent full-bridges was averaged to produce one calibrated compression measurement. The load cell was found have an accuracy of ±0.5N.

Paired scaphoids were tested sequentially with one scaphoid receiving an Acutrak 2 screw and the contralateral scaphoid received the Synthes screw. For all specimens, a retrograde 1.1 mm guide wire was inserted longitudinally and advanced under fluoroscopic control by an experienced surgeon (RG/KF). A longitudinal mark from the distal to proximal pole was then made prior to osteotomy to ensure post-osteotomy rotational alignment. The required screw length was measured, with the width of the load cell taken into account. The scaphoid was then predrilled/reamed under fluoroscopic guidance as per the manufacturer's instructions. With the guide wire removed, the scaphoid was secured to the cutting table with two Babcock clamps. A microsagittal saw was used to create an osteotomy perpendicular to the long axis of the scaphoid simulating a transverse waist fracture. The fracture was reduced with the load cell interposed between the fragments (Figure 3). Rotational alignment was confirmed, and the guide wire was reinserted before the appropriate screw was inserted. Each screw was advanced under fluoroscopic control until the operating surgeon judged that maximal compression had been obtained. The operating surgeon was blinded to the amount of compression measured by the load cell during insertion, in order to replicate intra-operative procedures.

The force of compression was continuously measured during screw insertion and continued for 180s after a steady state had been reached. Steady state was typically reached within 60-90s after peak compression was obtained, and was reached within 150s for all screws tested. The same procedure was repeated in the contralateral scaphoid using the comparison screw. Statistical analysis consisted of paired t-tests to compare the peak and final steady state compression for the two screw systems.

## Results

**Figure 4:**
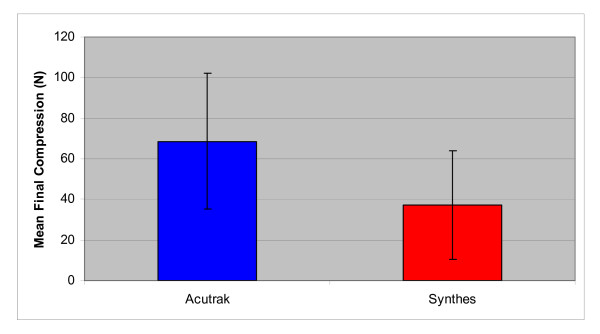
**The peak compression (±1 standard deviation) of the Acutrak and Synthes screws tested**.

**Figure 5:**
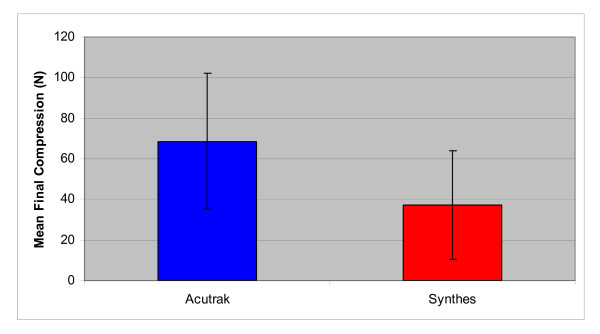
**The mean compression (±1 standard deviation) of the Acutrak and Synthes screws as measured from insertion to 180s after steady state**.

**Figure 6:**
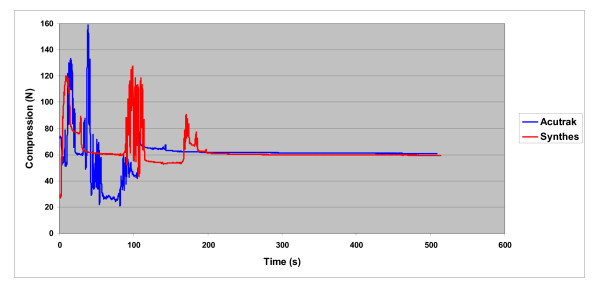
**A representative curve of the mean compression vs time for each screw system**.

The Acutrak 2 screw had higher measured peak and final interfragmentary compression than the Synthes screw, but this difference was only statistically significant in final compression. The mean peak compression (Figure 4) of the Acutrak 2 Standard was 103.9 ± 33.2N. Mean peak compression of the Synthes screw was 88.7 ± 38.6N (p = 0.13). The mean final compression (Figure 5) was 68.6 ± 36.4N for the Acutrak system, significantly higher than the Synthes screw which achieved 37.2 ± 26.8N of compression (p = 0.04). Throughout our trials a steady state was repeated reached with each screw system. This typically occurred after 120-150s. Representative curves are shown in Figures 6. A learning curve became apparent throughout the study. There was a failure with the first insertion of each screw set. Failure was characterized by a sudden loss of all compression. There were no fractures or screw breakage. These failures were likely due to incorrect measurement of the screw length secondary to the load cell. These two specimens were excluded from the data analysis and were not included in the sample size or statistical analysis. No further failures were observed with either system.

## Discussion

Our study has demonstrated that the Acutrak 2 screw system provides greater mean interfragmentary compression when compared to the Synthes headless compression screw in a static cadaveric scaphoid model. By using contralateral scaphoids to test each screw system, we were able to control for potential differences in bone quality between cadaveric subjects. In addition, the operating surgeon was blinded to the amount of compression generated. The surgeon was instructed to advance each screw based on tactile feedback and fluoroscopic imaging alone, replicating the intraoperative environment. While we found no statistical difference in the peak compression between the two systems, there was a statistically significant difference in the mean final interfragmentary compression (Acutrak 68.6 ± 36.4N vs. Synthes 37.2 ± 26.8N, p = 0.04).

Similar cadaveric studies have previously been reported. A study by Lo *et al *[18] found that the Synthes 3.0 mm headless compression screw, when used with a threaded washer generated a mean compressive value of 108 ± 60 N. This value is more than double the force generated in our tests. A possible explanation for this is that Lo *et al *[18] utilized a threaded washer which may have altered the tactile feedback generated by the screw/bone interface. Given that in both studies screws were advanced by feel, there is an inherent subjectivity that is difficult to control. Comparative methods [17] have been used to evaluate the Acutrak Standard, Acutrak Mini and Bold screws. This previous study found that the Acutrak Standard had a higher mean compressive force 5 min after installation than the comparative screws. Again, the compressive forces were markedly higher than in our study. Five minutes after compression, the compressive force for the Acutrak Standard, which is the predecessor of the Acutrak 2 Standard we tested, was 152 ± 21 N. Bailey *et al *[19] measured compressive values similar to those of our study, but the testing was done in a synthetic material. They achieved a mean compressive value of 38.8N with the Acutrak Standard screw. Overall then, there have been a wide range of the compressive values measured *in vitro*. Currently there is no consensus on the optimum interfragmentary compression needed to promote reliable bony union and limited conclusions can be made when comparing the absolute compressive forces generated across similar studies.

One possible reason that the Acutrak 2 screw provided greater final compression than the Synthes system may relate to the thread pattern. The Acutrak 2 is a fully threaded design that generates a large amount of thread-to-bone contact area. In comparison, the Synthes screw has fewer threads, which results in less thread-to-bone contact area, and thus greater stresses on the cancellous bone. The comparatively greater loss of compression experienced by the Synthes screw, may be evidence of the gradual failing of trabeculae due the higher stresses. Maximizing the number of effective threads may help to preserve post-insertion compression. This may mean the selection of screw length is less critical in the case of the Acutrak screws, as the fully threaded design may be less sensitive to minor errors in measured screw length. It should also be noted that the increased purchase afforded by the greater thread surface area, may make removal more difficult if required.

A limitation of this study is the age of the cadaveric specimens used. Although we did not formally assess bone quality, given the mean age of the specimens (75 years), we can infer that the bone quality was lower than what would be expected for the average patient with a scaphoid fracture. To address this issue, the matched pair design of this study helped to control for bone quality between the two screw systems.

## Conclusion

While our results suggest that the Acutrak 2 compression screw maintained greater compression than the Synthes screw, the clinical significance of this finding remains unclear. The literature has yet to demonstrate the amount of interfragmentary compression necessary to promote optimal fracture union. One can infer that reliable compression across the fracture site is required for predictable healing, and yet some degree of micromotion may also be advantageous. Further biological testing is needed to determine the correlation between compression, micromotion and bone biology. Additionally, the Synthes screw is a smaller screw and may cause less articular damage during and after insertion, thus leading to less post-operative pain and morbidity. Whether this is clinically significant is currently unknown and is an area of further investigation.

Our study has demonstrated that the Synthes and Acutrak 2 screws provide similar mean peak compression in a scaphoid fracture model, but the Acutrak 2 screw generates greater mean final interfragmentary compression. Before any clinical recommendations can be made, a clinical trial is necessary to determine if the identified differences in generated compression correspond to *in-vivo *differences in fracture healing.

## Competing interests

The authors declare that they have no competing interests.

## Authors' contributions

The lead author on this paper is RG. She is an experienced hand surgeon who performed the surgical techniques of the study and conceived the initial idea for the study and was responsible for revision of manuscript. The second author on the paper, JA, collected and analyzed the data and drafted the original manuscript. He is also the contact author. KF is an experienced hand surgeon who conceived and performed the study. He was also responsible for revision of the manuscript. DS, LF, and JJ were responsible for manuscript review, study conception and assisting with biomechanical issues associated with our study. All authors have read and approved the final manuscript prior to resubmission.
